# Validity of the Early Functional Ability scale (EFA) among critically ill patients undergoing early neurological rehabilitation

**DOI:** 10.1186/s12883-022-02855-3

**Published:** 2022-09-06

**Authors:** Melanie Boltzmann, Simone B. Schmidt, Christoph Gutenbrunner, Joachim K. Krauss, Günter U. Höglinger, Christian Weimar, Jens D. Rollnik

**Affiliations:** 1 BDH-Clinic Hessisch Oldendorf, Institute for Neurorehabilitation Research, Associated Institute of Hannover Medical School, Hessisch Oldendorf, Germany; 2 Department of Rehabilitation Medicine, Hannover Medical School, Hannover, Germany; 3 Department of Neurosurgery, Hannover Medical School, Hannover, Germany; 4 Department of Neurology, Hannover Medical School, Hannover, Germany; 5 Institute for Medical Informatics, Biometry and Epidemiology, University of Duisburg-Essen, Essen, Germany; 6 BDH-Clinic Elzach, Elzach, Germany

**Keywords:** Early functional abilities (EFA), Disorders of consciousness, Early neurological rehabilitation, Validity

## Abstract

**Background:**

A reliable assessment of the functional abilities of patients after severe brain damage is crucial for valid prognostication and treatment decisions, but most clinical scales are of limited use among this specific group of patients.

**Aim:**

The present study investigates the usefulness of the Early Functional Ability (EFA) scale, which determines the functional abilities of severely impaired patients.

**Methods:**

Critically ill patients consecutively admitted to early neurological rehabilitation were screened for eligibility. We assessed the correlation between the EFA scale and (i) the Early Rehabilitation Barthel Index (ERBI), and (ii) the Coma Recovery Scale-Revised (CRS-R). The 1-year outcome on the Glasgow Outcome Scale-extended (GOSE) was used to examine the predictive validity. Demographical and medical variables were entered into univariate and multivariate binary regression models to identify independent predictors of 1-year outcome.

**Results:**

Two hundred fifty-seven patients (168 men) with a median age of 62 years (IQR = 51–75) were enrolled. The correlation of the EFA scale with the CRS-R was high but low with the ERBI upon admission. Multivariate regression analysis yielded the vegetative subscale of the EFA scale as the only independent predictor for the 1-year outcome of patients admitted to early neurological rehabilitation.

**Conclusions:**

This study shows a high correlation of the EFA scale with the CRS-R but a weak correlation with the ERBI in patients with low functional abilities. With improving patient abilities, these correlations were partly reversed. Thus, the EFA scale is a useful tool to assess the functional abilities and the prognosis of critically ill patients adequately and may be more feasible than other scales.

## Introduction

Frequently, the functional abilities of patients undergoing neurological rehabilitation are assessed with instruments focusing on functional (in-)dependence in activities of daily living (ADL). The most widely used ADL scales are the „Functional Independence Measure “(FIM) [1] and the „Barthel Index “(BI) [2]. Both scales, however, are often not discriminative in patients with severe functional impairments. Patients with disorders of consciousness, for instance, score very low on ADL scales, although they may differ in their functional status and rehabilitation potential. In these cases, specific coma scales seem to be appropriate but show a ceiling effect once patients regain consciousness. Thus, critically ill patients undergoing early rehabilitation frequently have functional abilities that cannot be assessed on coma and ADL scales [3]. The Early Rehabilitation Index (ERI) was developed as an extension of the Barthel Index [4] to compensate for the disadvantage of ADL scales. The ERI consists of seven items addressing clinically significant aspects among patients admitted to early neurological rehabilitation. The BI and the ERI can be combined to the „Early Rehabilitation Barthel Index“, which shows better but not yet sufficient sensitivity for severely impaired patients. In addition, the „Early Functional Ability “(EFA) scale was developed to quantify more functional abilities among severely impaired patients and thus could be more sensitive for lower functional levels [5]. The EFA scale is mainly used in European rehabilitation centers (e.g., Germany [3], Denmark [6] and Norway [7]) due to the limited international availability of the manual and relevant publications. Thus, its validity so far could only be confirmed for the original German version [3] and a Danish translation [5]. In 2015 the EFA was fully translated into English [8], which may have contributed to a greater spread of the scale.

Since the EFA scale was developed to close the gap between patients with impaired consciousness and patients with better functions in ADL [3], it is of limited use for patients on higher functional levels. Therefore, some authors recommend combining different scales to compensate for the disadvantages of each scale. Two studies examining the feasibility of the EFA scale in patients with traumatic brain injuries conclude that the combination of different scales (e.g., EFA and FIM) might compensate for the shortcomings of each scale [5, 6]. This issue is relevant to early rehabilitation facilities since patients and their outcomes can be very heterogeneous [3] due to disorders of consciousness, the need of mechanical ventilation, and complications.

The present study aims to further investigate the validity of the EFA scale for critically ill patients with low functional abilities undergoing early neurological rehabilitation.

## Materials and methods

The study was conducted at the BDH-Clinic Hessisch Oldendorf, a large neurological rehabilitation center in Northern Germany. In Germany, the typical course of treatment for severe brain damage is described in a phase model: acute treatment (phase A), early rehabilitation (phase B), subsequent rehabilitation (phases C and D), occupational rehabilitation (phase E), and long-term care (phase F, offered in specialized nursing facilities). These phases do not necessarily have to be completed consecutively. Instead, it depends on initial disease severity, the recovery state, and the extent of regained abilities, which phases are passed. The present study focuses on phase B, which refers to the early, multimodal treatment of severely impaired patients requiring intensive care and monitoring. Frequently, those patients still have a tracheal cannula, need ventilatory support, suffer from disorders of consciousness, or have a higher risk for complications. Due to the heterogeneity, empirical therapy is administered depending on the individual functional and medical status. Patients enter subsequent rehabilitation phases once they can actively participate in treatments lasting 30 minutes or longer at least twice daily.

Five hundred two patients with severe brain damage, consecutively admitted to early rehabilitation (phase B) between June 2018 and February 2020, were screened for eligibility. Inclusion criteria were: (i) age above 18 years, (ii) first-ever stroke, hemorrhage, traumatic brain injury or hypoxic brain damage, and (iii) admission to an intensive care or intermediate care unit. Patients with other diagnoses (*n* = 142), incomplete data (*n* = 27), disease durations beyond 3 months (*n* = 5), EFA values ≥70 (*n* = 7), or who deceased during early rehabilitation (*n* = 38) were excluded from the study. Thus, 283 patients were enrolled in the study.

### Data collection

Demographic information (age, gender) and medical data (e.g., diagnosis, time since onset, functional status, and level of consciousness) were collected from medical records retrospectively. At the end of early rehabilitation, the length of stay and the type of discharge (e.g., nursing care, returning home) were determined. Finally, the Glasgow Outcome Scale-extended (GOSE) was assessed 1 year after discharge from early rehabilitation via telephone interviews (for further details, see [9]).

#### Functional status

The functional status was assessed using (i) the ERBI and (ii) the EFA scale. The ERBI consists of the BI and the ERI. The BI measures functional independence in activities of daily living through a panel of ten ordinal-scaled items resulting in a sum score of 0 to 100 (with 0 being completely dependent and 100 being completely independent). The ERI was introduced by Schönle [4] to record the following seven clinical criteria: (1) intensive care supervision, (2) tracheostomy tube management and supervision, (3) intermittent or continuous mechanical ventilation, (4) confusional state, (5) behavioral disturbances endangering oneself or others, (6) severe impairment of communication, and (7) dysphagia. For each criterion, a negative value of 25 points (communication disorder) or 50 points (all other criteria) is added to the sum score, resulting in a total score of 0 to − 325. Once the patient has overcome a certain condition, the respective value is removed from the score. The sum of the BI and the ERI results in the ERBI ranging from − 325 to 100, with lower values indicating higher impairment. A prerequisite for inclusion in early rehabilitation (phase B) is an ERBI score of ≤30. Since most patients admitted to early neurological rehabilitation are completely dependent on nursing, and the BI does not change over a long period, the ERBI is considered a useful additional assessment. The ERBI was evaluated once a week as part of the clinical routine by a team of nurses, therapists, and physicians. For this study, values upon admission and at the end of phase B treatment were used. The EFA scale comprises 20 items in four categories (Table 1): vegetative, oro-facial, sensorimotor, and cognitive functions. Each item was rated once upon admission using a five-point-scale (1 = no function, 2 = severe disturbance, 3 = moderate disturbance, 4 = slight disturbance, 5 = normal). Thus, EFA total scores may range from 20 to 100 [8]. Specific professions assessed the items of each subscale: vegetative functions by nurses, oro-facial functions by speech therapists, sensorimotor functions by physiotherapists, and cognitive functions by occupational therapists. The best response during the observation period over the first week was documented.

**Table 1:** Overview of the subscales and items of the Early Functional Abilities (EFA) scale [8]

Subscale	Item	Item Name
Vegetative	1	Autonomic stability
	2	Wakefulness
	3	Tolerance to postural changes
	4	Excretion functions (continence)
Oro-facial	5	Oro-facial stimulation/oral hygiene
	6	Swallowing
	7	Tongue movements/chewing
	8	Facial expression
Sensorimotor abilities	9	Muscle tone
	10	Head postural control
	11	Trunk postural control/sitting
	12	Changing position
	13	Standing
	14	Voluntary movements
	15	Locomotion/mobility in a wheelchair
Cognitive abilities	16	Tactile stimulation
	17	Visual stimulation
	18	Auditory stimulation
	19	Communication
	20	Comprehension

#### Consciousness

The level of consciousness was determined on the German version of the Coma Recovery Scale-Revised (CRS-R) [10]. The CRS-R consists of 23 hierarchically organized items divided into five functional subscales (auditory, visual, motor, oromotor/verbal, communication) and an arousal scale. The sum of the subscale values forms the total CRS-R score ranging between 0 and 23, with low values reflecting reflexive behavior and higher values indicating cognitively mediated behavior. The unresponsive wakefulness syndrome (UWS) is diagnosed when patients show either reflexive responses such as visual or auditory startle, localization of sounds, flexion withdrawal, abnormal posturing, oral reflexive movements, or no response. A minimally conscious state (MCS) is classified when the patient shows at least one of the following signs: consistent or reproducible movement to command, recognition or localization of objects, visual pursuit, fixation, automatic motor response, object manipulation, localization of noxious stimuli, intelligible verbalization, and non-functional intentional communication. Functional communication and/or functional object use indicate emergence from MCS (eMCS). CRS-R data were obtained during the first week after admission to the rehabilitation facility and at the end of phase B treatment. There are several scales to assess the level of consciousness after severe brain injury in clinical settings (e.g., the Glasgow Coma Scale [GCS] [11], the Full Outline of Unresponsiveness [FOUR] score [12], or the Wessex Head Injury Matrix [WHIM] [13]. The CRS-R was used in the present study because of its good sensitivy in discriminating patients in MCS from patients in UWS [14].

#### One-year follow-up

For the one-year follow-up, the outcome of surviving patients was assessed with the GOSE [15]. Therefore, individuals who have been specified as a caregiver during inpatient rehabilitation were contacted by phone. When the phone number was incorrect or the caregiver did not respond, an internet search was conducted to obtain further (contact) information. In a few cases, professional care facilities or professional guardians were contacted. The GOSE measures the outcome of brain injuries after discharge from inpatient treatment using an eight-point scale (1 = death; 2 = vegetative state, 3 = lower severe disability, 4 = upper severe disability, 5 = lower moderate disability, 6 = upper moderate disability, 7 = lower good recovery, 8 = upper good recovery). To determine the outcome category for each patient, the structured interview proposed by Wilson and colleagues [16] has been conducted.

### Statistical analyses

Data were analyzed with the Statistical Package for Social Sciences (SPSS; version 26) for windows. Differences were considered significant at a level of *p* < 0.05. For graphical representations, mean values and standard errors were used.

Since Shapiro-Wilk tests revealed that most continuous variables were not normally distributed (*p* < 0.05), non-parametric statistical methods were applied. Descriptive statistics are presented as median and interquartile range ([IQR], 25th and 75th percentiles). Differences between groups were analyzed using the Mann-Whitney U test (2 groups) or the Kruskal-Wallis test (> 2 groups) test. For categorical data, descriptive statistics are presented as frequencies. The Spearman correlation coefficient was used to analyze the correlation between different functional scales.

Binary logistic regression was used to investigate the predictive validity of the EFA subscales independent of other demographic and medical data. The GOSE score at 1-year follow-up was defined as the primary outcome measure, and a score > 3 was defined as a favorable outcome. In a first step, univariate analyses were performed with age, gender, time since onset of brain damage, traumatic vs. non-traumatic cause, and the functional status (ERBI, EFA, and CRS-R subscales) as independent variables. Subsequently, multivariate regression was performed (backward stepwise entry method [Wald]), including variables achieving *p* < 0.01 in univariate analysis. Odds ratios with the corresponding 95%-confidence intervals (CI) and explained variance (Nagelkerke’s R^2^) are reported. The model fit was assessed by means of the Hosmer and Lemeshow test for logistic regression, for which a *p*-value < 0.05 suggests a poor fit. The ability of the model to discriminate between patients with favorable and unfavorable outcomes at 1-year follow-up was quantified using the area under the receiver-operating characteristic curve (AUC).

## Results

### Patients

Two hundred eighty-three patients were enrolled in the study. Of these, 26 patients were lost-to-follow-up (9.19%) due to an incorrect phone number or refusal to provide outcome information. Thus, data of 257 patients were analyzed.

A description of the study sample is provided in Table 2. The median EFA score was 35 (IQR = 28–43), ranging between 20 and 68 points. While age and gender were not associated with the EFA sum and subscale scores, the EFA sum score (Z = 14.757; *p* = 0.002), as well as the sensorimotor (Z = 11.571; *p* = 0.009), the oro-facial (Z = 15.439; *p* = 0.001) and the cognitive (Z = 11.325; *p* = 0.010) subscales differed as a function of diagnosis (Table 2). Patients with hypoxic brain damage had lower sensorimotor, oro-facial, and cognitive abilities than those with other diagnoses (all *p* < 0.026). In addition, patients with ischemic stroke had higher cognitive abilities upon admission than patients with hemorrhage (Z = -2.180; *p* = 0.029).

**Table 2:** Patient characteristics

Variable	***n*** = 257	
Upon admission		
Age at event, years	62	(51 to 75)
Gender		
- *Male*	168	*(65.4%)*
- *Female*	89	*(34.6%)*
Time since onset, days	19	(12 to 28)
Diagnosis		
- *Ischemic stroke*	75	(29.2%)
- *Hemorrhagic stroke*	82	(31.9%)
- *Traumatic brain injury*	79	(30.7%)
- *Hypoxic brain damage*	21	(8.2%)
Early Functional Abilities	35	(28 to 43)
Early Rehabilitation Barthel Index	− 140	(− 170 to −90)
- *Early Rehabilitation Index*	−150	(−175 to −100)
- *Barthel Index*	10	(10 to 15)
Coma Recovery Scale-Revised	9	(4 to 15)
At discharge		
Length of stay, days	88	(55 to 112)
Early Rehabilitation Barthel Index	−10	(−85 to 30)
- *Early Rehabilitation Index*	−25	(−100 to 0)
- *Barthel Index*	15	(15 to 30)
Coma Recovery Scale-Revised	22	(11 to 23)
Type of discharge		
- *Professional care facility*	125	(48.6%)
- *Subsequent rehabilitation phase*	90	(35.0%)
- *Home care*	24	(9.3%)
- *Transfer to other facility*	18	(7.0%)

Values are frequencies (gender, diagnosis, type of discharge) or medians and interquartile ranges (all other variables)

#### Correlation analyses

(i) Early Rehabilitation Barthel Index.

**Fig. 1:**
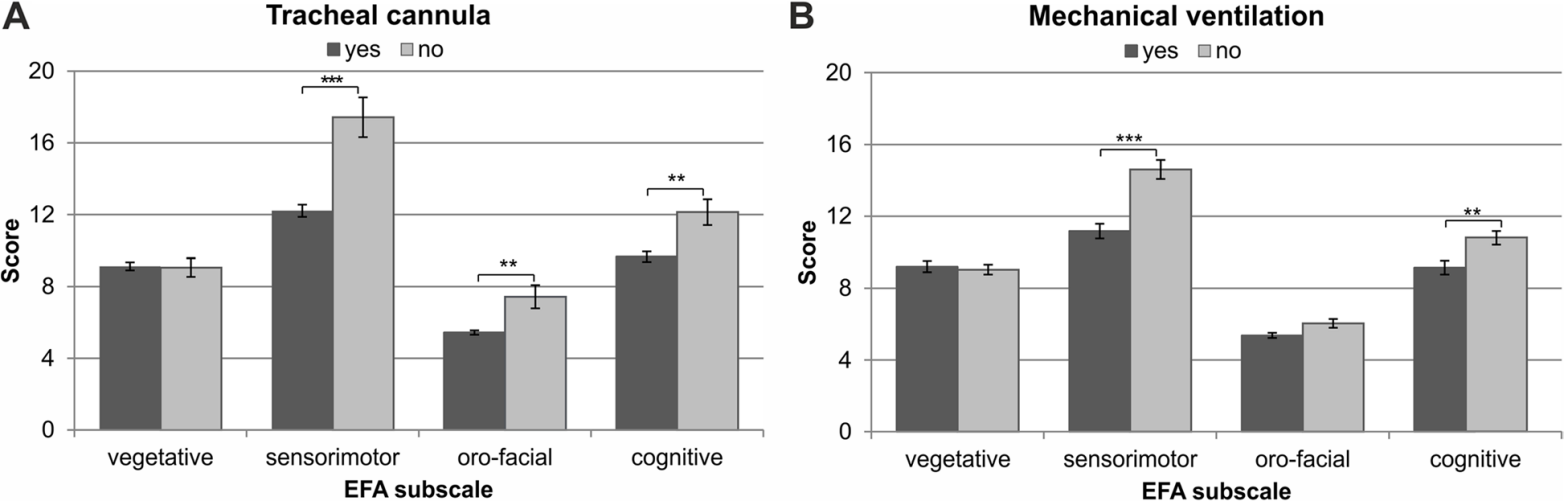
Differences in the EFA subscale scores as a function of **A**) tracheal cannula (ERI item 2) and **B**) mechanical ventilation (ERI item 3)

The median ERI and BI scores were − 150 (− 175 to − 100) and 10 (10 to 15), respectively. Upon admission, the correlation between EFA and ERBI was low (*r* = 0.118; *p* = 0.059). However, the higher the EFA total score upon admission, the higher the ERBI score at discharge (*r* = 0.515; *p* < 0.001). Especially, the presence of a tracheal cannula (ERI item#2) and the need for mechanical ventilation (ERI item#3) upon admission were associated with the EFA sum scale: Patients with a tracheal cannula (*n* = 227) had higher EFA scores than patients without a tracheal cannula (Z = -3.069; *p* = 0.002). The presence of a tracheal cannula was associated with the sensorimotor (Z = -4.194; *p* < 0.001), the oro-facial (Z = -2.681, *p* = 0.007), and the cognitive (Z = -2.853, *p* = 0.004) subscale (Fig. 1a). Likewise, patients on mechanical ventilation (*n* = 127) had lower EFA scores upon admission (Z = -2.735; *p* = .006). On the subscale level, the sensorimotor (Z = -4.544; *p* < 0.001) and the cognitive (Z = -2.891; *p* = 0.004) subscales were lower among these patients (Fig. 1b).

The higher the EFA total score, the higher the BI upon admission (*r* = 0.191; *p* = 0.002). The floor effect of the BI might explain the low correlation because the majority of patients (93.4%) had values< 20. No correlation was found between the EFA total score and the ERI upon admission and discharge.

(ii) Coma Recovery Scale-Revised.

The first CRS-R assessment classified 99 patients as UWS (38.5%) and 99 as MCS (38.9%). The remaining 59 patients (23.0%) were fully conscious. The CRS-R total score improved from 9 (IQR = 4 to 15) to 22 (IQR = 11 to 23) during rehabilitation (Z = -11.385; *p* < 0.001). At the end of early rehabilitation, 61 UWS patients (61.6%) and 73 MCS patients (73.7%) had improved in consciousness.

**Fig. 2:**
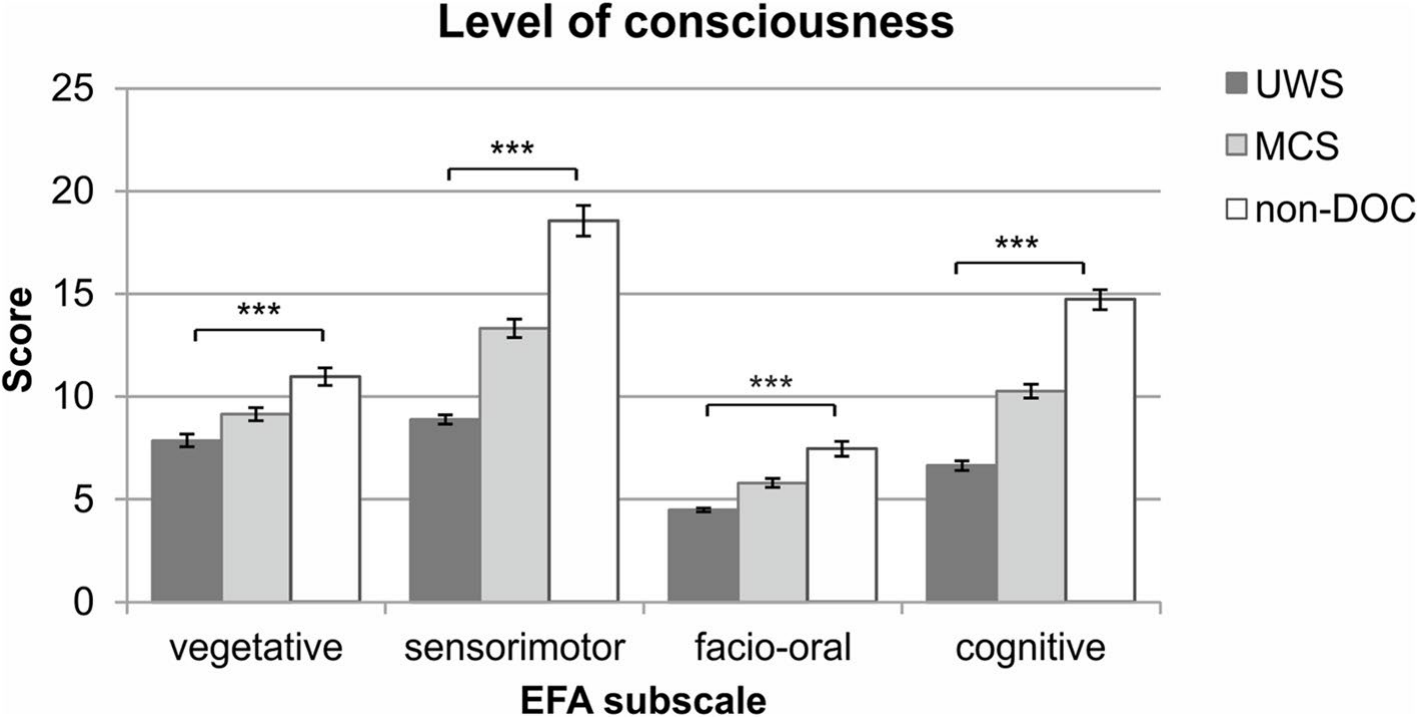
The EFA subscale scores stratified for different levels of consciousness upon admission

The EFA score was associated with the initial (*r* = 0.815; *p* < 0.001) and the final (*r* = 0.624; *p* < 0.001) CRS-R score. Subsequent analyses revealed that all CRS-R subscales correlated positively with the EFA total score and subscale scores upon admission and discharge. The EFA score was linearly associated with the level of consciousness (non-DOC > MCS > UWS); see Fig. 2.

### Predictive validity

**Fig. 3:**
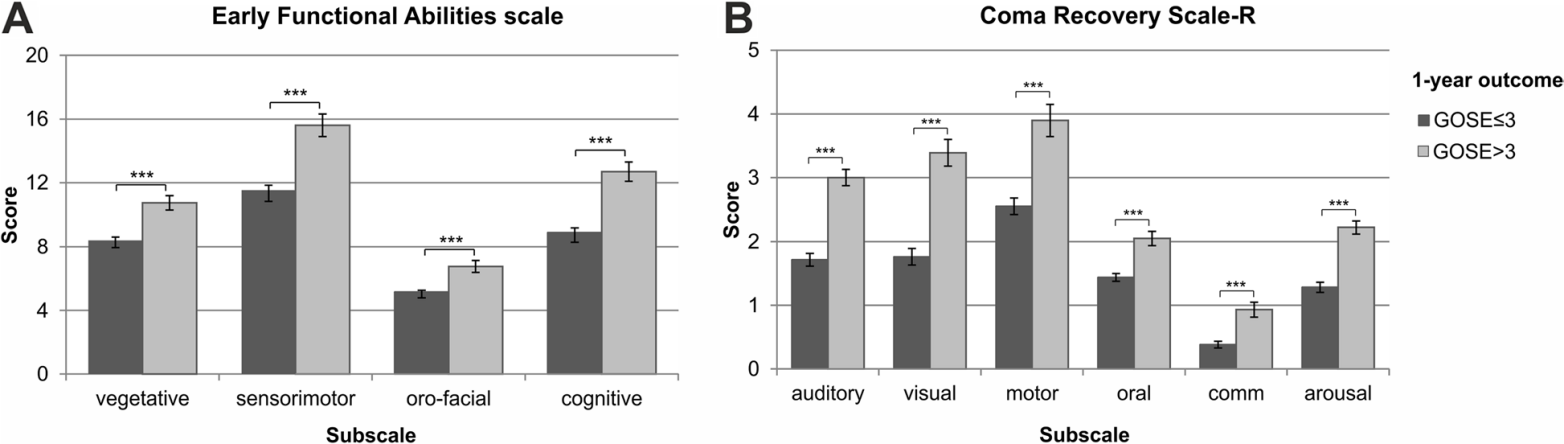
Differences in A) EFA and B) CRS- R subscale scores stratified between patients with favorable (GOSE> 3) vs. unfavorable (GOSE≤3) 1-year outcome

**Fig. 4:**
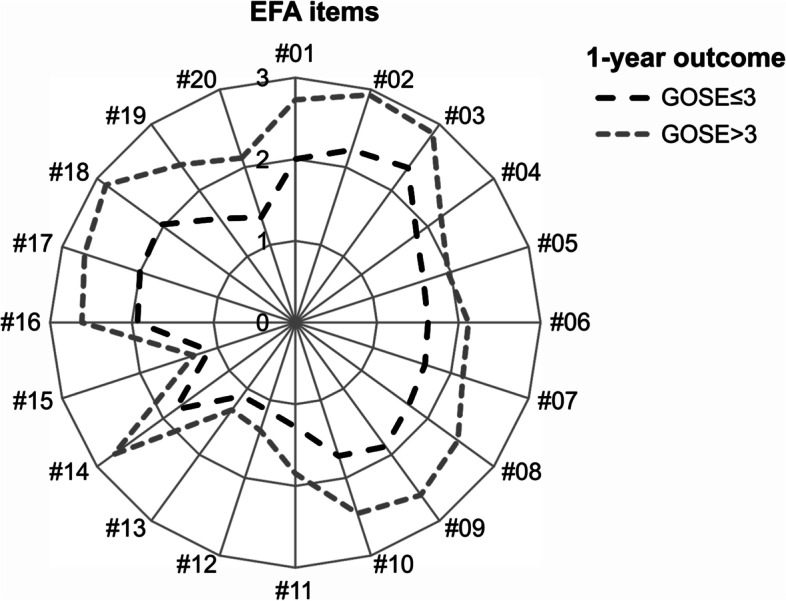
Differences in individual EFA items (*n* = 20) between patients with favorable (GOSE> 3) vs. unfavorable (GOSE≤3) 1-year outcome

At 1-year follow-up, 77.0% of patients (*n* = 198) had an unfavorable outcome, defined as GOSE≤3. Patients with a favorable outcome had higher values in the EFA total score (Z = -6.620; *p* < .001) and the four subscales (Fig. 3a). Subsequent analyses revealed that patients had lower scores in all individual EFA items (all *p* < 0.022; Fig. 4).

Likewise, patients with a favorable outcome had higher scores in the CRS-R subscales (Fig. 3b). In univariate analyses, factors associated with the 1-year-outcome were age, time since onset, length of stay in early rehabilitation, traumatic cause, and functional status upon admission (i.e., BI, EFA, and CRS-R scores), see Table 3. These factors were included as independent variables in the multivariate analysis.

**Table 3:** Univariate (unadjusted) and multivariate (adjusted) logistic regression for predictive factors of favorable outcome (GOS > 3)

Independent variable	Unadjusted			Adjusted		
	OR	CI 95%	*p*	OR	CI 95%	*p*
Age	**0.97**	**0.95–0.98**	**< 0.001**	**0.94**	**0.92–0.97**	**< 0.001**
Male gender	1.57	0.83–2.98	0.169			
Time since onset	**0.96**	**0.93–0.99**	**0.004**	**0.95**	**0.91–0.98**	**0.014**
Traumatic cause	**2.60**	**1.43–4.75**	**0.002**	**4.48**	**1.85–10.89**	**0.005**
Admission to ICU	0.94	0.53–1.80	0.936			
Length of stay	**0.97**	**0.96–0.98**	**< 0.001**	**0.97**	**0.96–0.99**	**< 0.001**
*ERBI*						
- BI	**1.12**	**1.05–1.20**	**0.001**			
- ERI	1.00	0.99–1.00	0.565			
*EFA subscales*						
- Vegetative	**1.25**	**1.14–1.37**	**< 0.001**	**1.21**	**1.06–1.38**	**0.004**
- Sensorimotor	**1.17**	**1.10–1.25**	**< 0.001**			
- Facio-oral	**1.45**	**1.24–1.70**	**< 0.001**			
- Cognitive	**1.24**	**1.15–1.33**	**< 0.001**			
*CRS-R subscales*						
- Auditory	**2.16**	**1.65–2.84**	**< 0.001**	**1.99**	**1.39–2.86**	**< 0.001**
- Visual	**1.60**	**1.35–1.89**	**< 0.001**			
- Motor	**1.44**	**1.23–1.68**	**< 0.001**			
- Oral	**2.28**	**1.59–3.28**	**< 0.001**			
- Communication	**2.09**	**1.50–2.92**	**< 0.001**			

The multivariate analysis provided the following factors independently associated with a positive outcome: young age (OR = 0.94 [95% CI: 0.91–0.97]; *p* < 0.001), early admission to the rehabilitation facility (OR = 0.94 [95% CI: 0.90–0.98]; *p* = 0.007), traumatic cause (OR = 3.95 [95% CI: 1.52–10.24]; *p* = 0.005), short length of stay in early rehabilitation (OR = 0.97 [95% CI: 0.96–0.99 *p* < 0.001) as well as higher scores in the vegetative EFA subscale (OR = 1.23 [95% CI: 1.06–1.42 *p* = 0.005) and the auditory CRS-R subscale (OR = 2.48 [95% CI: 1.30–4.76 *p* = 0.013) upon admission. The Hosmer-Lemeshow test indicated an adequate goodness of fit (*p* = 0.676). Together, the predictors accounted for 61% of variability in the model (Nagelkerke’s R^2^ = 0.614), and AUC was 0.924 (95% CI [0.883–0.965]).

## Discussion

The present study investigated the correlation of the EFA scale with other scales and the predictive validity for the 1-year outcome of critically ill patients undergoing early neurological rehabilitation. A reliable assessment of the functional status is crucial in the acute phase after the onset of the brain damage but is still of importance during early rehabilitation. Different scores have been established for the clinical routine, each associated with specific advantages and disadvantages. The EFA scale has been proposed as an addition to the existing ADL and coma scales.

### Correlation with other scales

Upon admission to early rehabilitation, no association between the ERI and the EFA sum score was found. The ERI items describe the severity of the brain damage and how long it takes until the patient has overcome critical conditions (e.g., has successfully been weaned from mechanical ventilation or the tracheal cannula). However, due to dichotomous scaling in “present” vs. “not present”, the ERI is rather imprecise, resulting in a lack of sensitivity. Further graduations of each item would allow a more detailed evaluation of the patient’s abilities. In contrast to the ERI, the EEA scale does not focus on the need for intensive monitoring, mechanical ventilation, or a tracheal cannula. In addition, the items used are more differentiated than the ERI items, which might explain the missing correlation between both scores.

Although the second component of the ERBI, the BI, correlated with the EFA sum scale, the correlation coefficient suggested only a weak relationship. Since the BI is known to have a floor effect, it can be assumed that this scale is not suitable to depict the functional abilities of patients with severe impairments adequately. Unlike the BI, the EFA scale is not based on complex activities relevant for independence in everyday life like the BI. It records the early functional abilities and clinically observable changes regarding situation-specific activities, which in turn promote the ability to actively participate in therapeutic interventions [3]. In addition, the EFA compensates for another weakness of the ERBI because cognitive functions are also addressed. The higher correlation at the end of rehabilitation suggests that the ADL scales become more valuable at later time points when patients have achieved higher functional abilities. The low correlation between the EFA and the ERBI scale upon admission suggests that the EFA scale is a useful addition to ADL scales.

In contrast to the ERBI, there was a high correlation with the CRS-R, an internationally established scale used to evaluate disorders of consciousness. The high correlation between both scales is partly due to the considerable overlap in content. For example, both instruments use items focusing on auditory, visual, oromotor, sensorimotor/tactile, and communicative abilities. In addition, many patients admitted to early neurological rehabilitation suffer from disorders of consciousness upon admission, which might contribute to the high correlation between both scores. This hypothesis is confirmed by the fact that the correlation declines with increasing functional abilities that are often accompanied by an improved consciousness. However, the EFA scale focuses on different aspects of functional recovery and identifies areas where rehabilitation efforts are particularly required [3]. Thus, the EFA scale accounts for clinical conditions in more detail than the CRS-R scale. Moreover, the EFA scale goes beyond the CRS-R scale by assessing vegetative functions, including autonomic stability, alertness, tolerance to postural changes, and continence.

A primary limitation of the CRS-R scale is that no further progress can be recorded once the patient has fully regained consciousness. Since the EFA scale was only collected once at the beginning of rehabilitation, a further study should be conducted with repeated measurements, e.g., after 4 weeks and at the end of early rehabilitation, in order to examine how the correlation with the CRS-R scale changes over time.

### Predictive validity

In univariate analyses, the 1-year outcome was predicted by the patient’s age, time since onset, traumatic cause, length of stay, BI, and the individual EFA and CRS-R subscales. In the multivariate regression model, age still predicted the functional outcome, with younger patients being more likely to have a favorable outcome. Age has repeatedly been reported to be an essential outcome predictor in patients with severe brain damage [17–20]. A possible explanation might be that elderly patients exhibit higher morbidity and altered brain plasticity, which may influence their ability to recover after brain damage [21, 22]. Another important factor associated with the outcome was the traumatic cause of the disease. More specifically, patients with traumatic brain injuries were more likely to have a favorable outcome than patients with non-traumatic brain injuries (e.g., stroke, hemorrhages, and hypoxic brain damage). This result is in line with previous studies showing that traumatic injuries are associated with better outcomes than non-traumatic injuries [17, 18, 23, 24]. However, it must be noted that patients with traumatic etiologies are often younger than patients with non-traumatic damages. Therefore, it is reasonable to assume that their better outcome might be related to their younger age rather than the traumatic causation. Two other factors associated with the outcome were the length of stay (LOS) in acute care hospitals before they were admitted to early rehabilitation and the LOS in early rehabilitation. In both cases, a shorter LOS has been proven to be beneficial for the outcome 1 year after discharge from early rehabilitation. This relationship is probably caused by the initial severity of the disease because patients with less severe brain damage might be stabilized faster and therefore submitted earlier to the rehabilitation facility. Likewise, patients with lower morbidity may have a shorter LOS in early rehabilitation because they are transferred to subsequent rehabilitation phases once being fully conscious, cooperative, and able to actively participate in therapies. It can be assumed that less impaired patients functionally improve more quickly than more affected patients.

Only the auditory CRS-R subscale proved to be an independent predictor of the 1-year outcome, with patients showing higher auditory abilities upon admission having a better chance of a positive outcome. The auditory scale of the CRS-R includes not only basic auditory processing (e.g., auditory startle reflex or orientation toward the location of sounds) but also reproducible and/or consistent movements to commands. In addition to perceiving and processing auditory information, command following requires the understanding of language and the ability to convert the instruction into a motor response. This issue has also been pointed out as a limitation of the CRS-R scale since the administration of the scale is based on the assumption that the items are hierarchically structured and that all items of one subscale rely on the same neurological structures [25]. However, Sattin and colleagues [26] argue that this might not apply to the items used in the CRS-R subscales. The response to tones, for example, is most likely processed by other neuronal structures than those responsible for cognitively mediated motor responses. Hence, the demands on cognitive functions are much more complex in the auditory subscale of the CRS-R scale than the one of the EFA subscale, where unspecific/specific reactions to auditory stimulation (tones, familiar/unfamiliar voices) are recorded.

Among the EFA subscales, vegetative functions proved to be an independent predictor for the 1-year outcome. Since this subscale is quite heterogeneous, each item will be discussed individually below. The first item of the vegetative subscale, „autonomic stability“, has been previously shown to predict the outcome. In patients with disorders of consciousness, for example, dysfunctions of specific parameters of the autonomic nervous are associated with a poorer outcome and the extent of reduced consciousness [27]. The second item focuses on the wakefulness of patients, discriminating patients with no, infrequent, and regular sleep-wake cycles. Several studies have reported that severe brain damage profoundly affects sleep patterns and the sleep-wake cycle (e.g., [28, 29]). The observed sleep disturbances are a prognostic marker for outcome [28] and can be used to discriminate UWS from MCS early after brain damage [30]. Overall, the sleep patterns after acute brain damage are often related to the extent to which brain structures and pathways involved in arousal and cognition are damaged [31]. The item “tolerance to postural changes” is a recommended standard of care in patients with severe brain damage to establish functional mobility. Postural changes are one part of physical treatment interventions in DOC patients, although the effectiveness has not yet been confirmed. However, physical management strategies including postural control techniques, range of motion exercises, postural control techniques, skincare, nutritional management, sensory stimulation, and restoration of sleep-wake cycles increased the incidence of recovery 1-year post-onset [32]. The last item was „continence“, which showed a floor effect since the majority of patients included in the study were provided with a urinary catheter system or used urine bottles. However, because only severely impaired patients (admitted to ICU or IMC) were included, there might be more heterogeneity in other clinical samples. Urinary functions should be further investigated in future studies since urinary incontinence has been shown to be associated with functional recovery and survival in stroke patients [33].

## Conclusion

Prognosticating the short- and long-term outcomes of patients with severe brain damage is essential during the treatment process. Therefore, clinical scales are necessary, which can reliably assess the functional status and monitor longitudinal changes of critically ill patients. The present study investigated the validity of the EFA scale in patients with particularly low functional abilities. While there was a high correlation with the CRS-R scale upon admission to early rehabilitation, the association declined during rehabilitation. In contrast, no significant correlation between the EFA and the ERBI was found at the beginning but at discharge from early rehabilitation. These results indicate that the EFA scale should be considered to be implemented in acute and early rehabilitation phases as a standard assessment because it covers a specific range of functional abilities, which are not properly part of coma and ADL scales.

### Limitations

The German model of neurological rehabilitation differs from other countries because some patients entering early rehabilitation are still comatose and mechanically ventilated. In other countries, these patients might not be eligible to enter rehabilitation and would rather stay in an ICU of acute-care hospitals. In addition, post-acute rehabilitation is offered for all kinds of neurological and neurosurgical disorders (hypoxic, traumatic, vascular, and other) “under one roof” instead of more specialized centers. This might also explain why the median age of the present study sample is higher than in other studies focusing on traumatic brain injuries. These differences might limit the transferability of the results to younger patients and to other countries with different healthcare systems.

In addition, the study sample was restricted to patients who were admitted to intensive or intermediate care units, although early neurological rehabilitation also includes patients admitted to peripheral wards who may have higher functional abilities. Since the results of the present study are not directly generalizable to all patients entering early rehabilitation, future studies should be conducted with samples representing the whole group of patients admitted to early neurological rehabilitation.

## Data Availability

The datasets supporting the conclusions of this article are available from the corresponding author on reasonable request.

## Abbreviations

ADL: Activities of daily life
AUC: Area under the receiver-operating characteristic curve
BI: Barthel Index
CI: Confidence interval
CRS-R: Coma Recovery Scale-Revised
EMCS: Emergence from MCS
EFA: Early Functional Abilities
ERBI: Early Rehabilitation Barthel Index
ERI: Early Rehabilitation Index
FIM: Functional Independence Measure
GOSE: Glasgow Outcome Scale-Extended
IQR: Interquartile range
MCS: Minimally conscious state
OR: Odds ratio
UWS: Unresponsive wakefulness syndrome
